# Dietary Acrylamide and the Risks of Developing Cancer: Facts to Ponder

**DOI:** 10.3389/fnut.2018.00014

**Published:** 2018-02-28

**Authors:** Jaya Kumar, Srijit Das, Seong Lin Teoh

**Affiliations:** ^1^Department of Physiology, Faculty of Medicine, Universiti Kebangsaan Malaysia Medical Centre, Kuala Lumpur, Malaysia; ^2^Department of Anatomy, Universiti Kebangsaan Malaysia Medical Centre, Kuala Lumpur, Malaysia

**Keywords:** acrylamide, food, nutrition, cancer, mechanism, human

## Abstract

Acrylamide (AA) is a water soluble white crystalline solid commonly used in industries. It was listed as an industrial chemical with potential carcinogenic properties. However to date, AA was used to produce polyacrylamide polymer, which was widely used as a coagulant in water treatment; additives during papermaking; grouting material for dams, tunnels, and other underground building constructions. AA in food could be formed during high-temperature cooking *via* several mechanisms, i.e., formation *via* acrylic acid which may be derived from the degradation of lipid, carbohydrates, or free amino acids; formation *via* the dehydration/decarboxylation of organic acids (malic acid, lactic acid, and citric acid); and direct formation from amino acids. The big debate is whether this compound is toxic to human beings or not. In the present review, we discuss the formation of AA in food products, its consumption, and possible link to the development of any cancers. We discuss the body enzymatic influence on AA and mechanism of action of AA on hormone, calcium signaling pathways, and cytoskeletal filaments. We also highlight the deleterious effects of AA on nervous system, reproductive system, immune system, and the liver. The present and future mitigation strategies are also discussed. The present review on AA may be beneficial for researchers, food industry, and also medical personnel.

## Introduction

Acrylamide or 2-propenamide (AA, C_3_H_5_NO) is a water soluble white crystalline solid with a relative molecular mass of 71.08 kDa, commonly used in industry (1). In the year 1994, the International Agency for Research on Cancer listed AA as industrial chemicals with potential carcinogenic risk to humans (1). However to date, AA was used to produce polyacrylamide polymer, which remained widely used as a coagulant in water treatment; additives during papermaking; grouting material for dams, tunnels, and other underground building constructions; and as electrophoresis gels (2–5).

## Formation of AA in Food Products

Later in the year 2002, AA was detected in heated foods where its formation was temperature dependent (6). Using liquid chromatography–mass spectrometry, moderate levels of AA (5–50 µg/kg) were detected in heated protein-rich foods, but higher contents of AA (150–4,000 µg/kg) were detected in carbohydrate-rich foods (6). Importantly, AA could not be detected in unheated or boiled foods (6). AA in food could be formed during high-temperature cooking *via* several mechanisms, i.e., formation *via* acrylic acid which may be derived from the degradation of lipid, carbohydrates, or free amino acids; formation *via* the dehydration/decarboxylation of organic acids (malic acid, lactic acid, and citric acid); and direct formation from amino acids (7).

Studies have shown that AA is formed mainly from free amino acid asparagine and reducing sugars such as glucose and fructose, during high-temperature cooking through Maillard reactions, a series of non-enzymatic reactions between free amino acids and reducing sugars which is responsible for the flavor and color generated during baking (8, 9). This would explain the formation of AA in cooked food rich in asparagine, e.g., in cereals and potatoes (8). In fact, the concentration of reducing sugars in food is the primary determinant of AA formation, compared to asparagine content (10).

The mean AA contents in potato crisps prepared from different UK-grown potatoes ranged from 131 to 5,360 µg/kg (11). Rosti, a popular Swiss dish, made of grated and fried potatoes, contains an average of 702 µg/kg AA (12). The AA concentration in different commonly consumed breads ranges from <limit of quantification (LOQ) to 695 µg/kg, where the highest AA concentration was detected in wheat bran and whole wheat breads (13). Similarly, AA was also detected in different bakery products: biscuits (LOQ to 2,405.0 µg/kg), sandwich biscuits with cream (112.6–570.4 µg/kg), gingerbread (349.5–955.5 µg/kg), and crackers (347.8–366.1 µg/kg) (14). In addition to potato- and cereal-based food, coffee beverages acquired from coffee vending machines also contain AA concentration which varies from 7.7 to 40.0 µg/L (15).

Estimated AA intakes were measured using food frequency questionnaire, total diet study, hemoglobin adducts of AA, and glycidamide (GA, primary metabolite of AA) (16, 17). For adults, the estimated average AA intakes range from 0.3 to 0.6 µg/kg of body weight per day, where the intake is higher in children (18).

Infant cereal-based foods are important nutrient source for children around the world, and the AA concentration in these cereal-based baby food (ready-to-eat and instant baby foods, candy bars, and cakes) varied between 10 and 60 µg/kg (19). In another study, the mean AA level in cereal-based baby foods ranged from 36 to 604 µg/kg, and it was estimated the mean AA exposures of toddlers from the cereal-based baby food was 1.43 µg/kg of body weight per day (20).

## AA Consumption and Cancer

High-income countries have the highest incidence rates for lung, colorectal, breast, and prostate cancers, while low- and middle-income countries have the highest rates of stomach, liver, and esophageal and cervical cancer (21). Global Burden of Disease Cancer Consortium et al. (22) has reported that there were a total of 17.5 million cancer cases globally, 8.7 million deaths, and 208.3 million disability-adjusted life-years, in the year 2015. Between the year 2005 and 2015, incident cancer cases were found to be increased by 33% (22).

Since the discovery of AA in foods, numerous studies have set to explore the carcinogenic potential in humans. Although AA was shown to be carcinogenic in both male and female rodent models, numerous studies reported no statistically significant association between dietary AA intake and various cancers in humans, e.g., pancreatic, prostate, breast, ovarian, and endometrial cancer (23–30). Obon-Santacana et al. (26) analyzed the AA and GA hemoglobin adduct levels in quintiles based on control distributions and showed no effect on overall endometrial cancer risk (HR_HbAA;Q5vsQ1_: 0.84, 95% CI: 0.49–1.48; HR_HbGA;Q5vsQ1_: 0.94, 95% CI: 0.54–1.63). Similarly, no clear association were shown between AA intakes and the risk of epithelial ovarian cancer, except some positive associations were observed between second (59.81–78.70) and third (78.80–106.00) quintiles of total AA and GA hemoglobin adduct levels (OR_Q2vsQ1_, 1.81; 95% CI: 1.06–3.10) and (OR_Q3vsQ1_, 2.00; 95% CI: 1.16–3.45) (27). Interestingly, Hogervorst et al. (31, 32) showed significant interaction between AA intake and single-nucleotide polymorphisms (SNPs) in AA-metabolizing enzymes cytochrome P450, family 2, subfamily e, polypeptide 1 (CYP2E1) with endometrial cancer risk, and SNPs in the HSD3B1/B2 gene cluster (through effects on progesterone or androgens) with ovarian cancer risk.

Few studies reported possible association between dietary AA intake and cancer risk, e.g., dietary AA may increase the risk for cutaneous malignant melanoma in men (HR: 1.13, 95% CI: 1.01–1.26) per 10 µg increment (33). Similarly, dietary AA may also increase the risk of lymphatic malignancies (i.e., multiple myeloma and follicular lymphoma) in men (34). The adjusted risk of all esophageal tumors combined among Sweden esophageal cancer patients was in the highest quartile of AA intake compared to the lowest, particularly among overweight or obese individuals (35). However, in the European Prospective Investigation into Cancer and Nutrition (EPIC) cohort, an association between dietary AA intake and an increased risk of developing esophageal cancer was observed in the middle quartiles, not in the highest quartile (36). A recent study revealed dietary AA intake was associated with increased overall cancer mortality in elderly Chinese (37). When compared to the lowest (<9.9 μg/day) and the highest (>17.1 μg/day) quartile of AA intake, the multivariable hazard ratios were 1.9, 1.9, and 2.0 for the overall, digestive, and respiratory system cancer mortality, respectively (37).

## Body Enzymatic Influence on AA

In human and animals, AA is primarily metabolized to an epoxide metabolite, GA, by an enzyme known as CYP2E1 (38, 39). GA may subsequently conjugated with reduced glutathione (GSH) to GSH conjugates by GSH transferases, or GA also can be further metabolized to glyceramide by epoxide hydrolase.

## GA Product of CYP2E1: Relevance in Potential Carcinogenic Effect of AA

Using CYP2E1-null and wild-type mice, Sumner et al. (40) showed that the absence of CYP2E1 enzyme in the mutant mice led to non-excretion of GA or its metabolites in the urine of ^13^C-AA-treated mice. Subsequent studies by Ghanayem et al. (39) showed that CYP2E1-mediated oxidation of AA to GA is required for the induction of male germ cell mutagenicity in mice exposed to AA. In their subsequent study, the researchers went further to show that GA through interaction with DNA in the testes/sperm or spermatid protamine formed DNA adducts that leads to male germ cell mutagenicity. In a recent study, Ehlers et al. (41) investigated the effect of low and high doses of GA (1–0.0001 mM) on the expression of genes involved in cancer development using human ovarian and endometrial cancer cell lines and human primary hepatocytes. The authors reported high dose of GA (1 mM) to upregulate the genes involved in oncogenesis such as TF3 (ovarian and endometrial cell lines), dual specific phosphatases DUSP1, DUSP4, and DUSP5 (ovarian cell lines), zinc finger protein gene ZNF746, cYMC, and Bystin-like protein gene (BYSL) (primary human hepatocytes) (41).

## GSH, GST, and AA

GSH is an important antioxidant that prevents cellular damage caused by reactive oxygen species such as peroxides, free radicals, heavy metals, and lipid peroxides (42). GST is a group of enzymes that mainly involved in detoxifying xenobiotics, cell death, and cell proliferation (43). GSH is employed by GST in numerous cellular reactions. Thus, the enzymatic activities of GST depend upon the availability of GSH. Srivastava et al. (44) investigated the effect of single and repeated administration of AA on GSH content and GST activity in rat brain. The researchers reported single administration of AA to deplete GSH content without affecting GST activity. However, repeated administration of AA (50 mg/kg × 10 days) depleted GSH content as well GST activity in the rat brain. Altered GST activity may affect the redox status of the cell. This may subsequently affect various redox-dependent gene expression, cell transformation or proliferation, or even apoptosis may occur (45–48).

## Hormonal Mechanism of Action of AA in Thyroid Tumor in Male Rats

**Figure 1:**
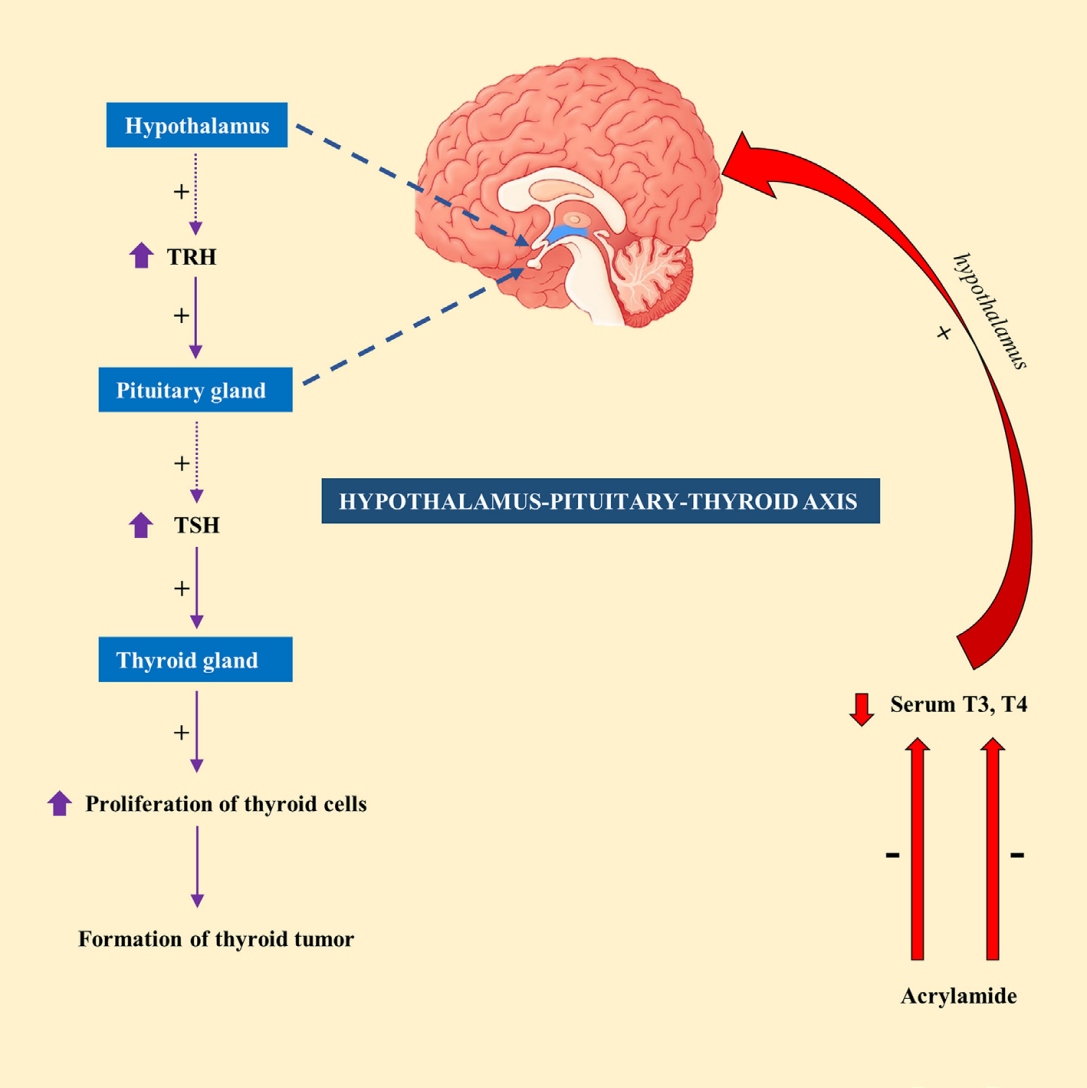
A schematic representation showing the mechanism of action of acrylamide (AA) in thyroid tumor in rats. Exposure to AA reduces serum T3 and T4, which stimulate hypothalamus of hypothalamus-pituitary-thyroid axis to secrete thyrotropin-releasing hormone (TRH). TRH stimulates pituitary gland to release thyroid-stimulating hormone (TSH), which subsequently stimulate thyroid gland to initiate proliferation of cells and eventually development of thyroid tumor.

Exposure of male F344 rats to AA (50 mg/kg) in drinking water for 14 days resulted in a significant decrease in serum T4 level (49). In contrary, exposure to lower doses of AA (2.5 or 15 mg/kg) had no significant effect on serum T4 or thyroid-stimulating hormone (TSH) level in male F344 rats (50). This led to the suggestion that hormonal mechanism is likely to attribute to thyroid tumorigenesis, at least at high doses of AA (51). Hormonal mechanism is thought to be influenced by decreased circulating levels of T3 or T4 levels, which would subsequently stimulate the release of TSH by the pituitary gland *via* hypothalamus–pituitary–thyroid axis. Continuous stimulation of the thyroid gland by TSH results in increased proliferation of cells and so the formation of tumor (52). A schematic diagram showing the mechanism of action of AA in thyroid tumor was highlighted in Figure 1.

## Calcium Signaling—Cytoskeletal Mode of Mechanism

**Figure 2:**
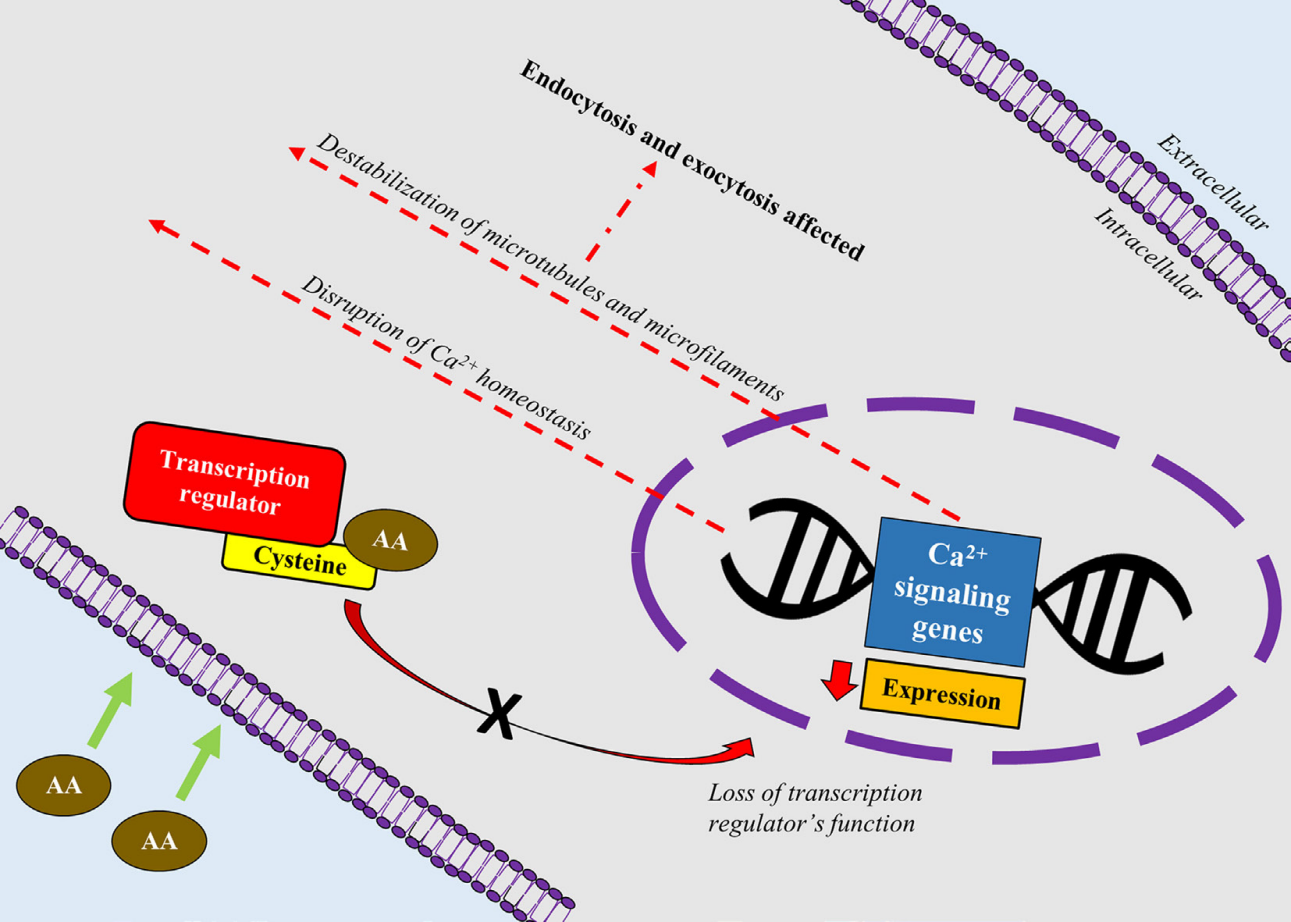
A schematic depiction correlating calcium signaling and cytoskeletal filaments to genotoxic effects of acrylamide (AA) in rats’ testes. AA binds toward redox-sensitive Cys residues on key regulatory proteins such as transcription factors, which disrupts the protein’s function. Loss of the transcription regulator’s function causes reduced expression of calcium signaling genes and disruption of calcium homeostasis. Reduced expression of calcium signaling genes also destabilizes microtubules and microfilaments which carry out endo- and excytotic functions.

A recent study correlated calcium signaling and cytoskeletal filaments to genotoxic effects of AA in testes using F344 rats (53). Similar to this finding, existing literature implicates calcium signaling with several types of cancers (54). Binding of AA toward redox-sensitive Cys residues on key regulatory proteins initiate disruption of calcium signaling genes which could lead to reduced expression of calcium signaling genes and disruption of calcium homeostasis. Reduced expression of calcium signaling genes may destabilize microtubules and microfilaments which carry out endo- and excytotic functions (Figure 2). It is hypothesized that disruption in calcium signaling also could lead to cancer development *via* hormonal actions. However, lack of empirical proof warrants his hypothesis to be further investigated (55).

## Cytoskeletal—AA—Genotoxic Effects

The idea of cytoskeletal proteins mediating the genotoxic effects of AA were implicitly corroborated using various study models (55, 56). Cytoskeletal proteins are tasked with multitude of functions that integral for cell survival such as movement of vesicles and cell compartments, regulation of cell division (57), and cell mechanics (58). Few studies reported AA to bind directly to cytoskeletal proteins (59–61). Given the direct functional relation between the cytoskeletal proteins and cell divisions (mitosis/meiosis), AA’s disruptive effects pose various adverse effects, including delays in cell cycle (62), heritable translocation of chromosomes (63), chromosomal aberrations (64), blocks in mitotic and meiotic process (59, 65), and aneuploidy (66).

The primary targets of AA are kinesin motors which are proteins critically involved in cell divisions (67). Kinesins are motor proteins that move along microtubules, fueled by hydrolysis of ATP. Movement of kinesins aids various cellular functions including the transport of cellular cargo from central toward cell periphery. As tailored to their functions, the most common feature of kinesin is presence of motor domain for microtubule binding and ATPase activities. Kinesins are categorized as N (N-terminal), C (C-terminal), and M or I-type (middle or also known as non-motor) based on the location of motor domain within the macromolecule (68). AA was shown to inhibit kinesin in neurons (60) and testes (61). In 2007, Sickles and colleagues investigated the effects of AA, glycidamide (AA’s toxic metabolite), and propionamide (non-neurotoxic metabolite) on function of specific kinesins, KIFC5A (member of kinesin-14 subfamily with motor domain located at the carboxy terminus of the polypeptide), and KRP2 (member of kinesin-12 subfamily with motor domain located more toward the center of the polypeptide). Generally, KIFC5A stabilizes spindle pole by crosslinking the adjacent microtubules (69, 70) and KRP2 regulates microtubule dynamics and chromosome segregation during anaphase (71). The researchers reported AA and glycinamide to specifically inhibit both KIFC5A and KRP2 kinesins (61). Their findings were similar to their previous findings where AA and glycinamide inhibited neuronal N-type kinesins (60). Despite their findings which were convincing at *in vitro* level, the authors concluded that the carcinogenicity of AA and glycinamide were inconclusive and this warrants future studies to further elucidate the conundrum.

## Other Harmful Effects of AA on Body

### Neurotoxic Effects of AA

The neurotoxic effects of AA is the only toxic effect proven to be harmful on humans *via* occupational exposure (72, 73). The clinical signs of AA neurotoxicity were peripheral neuropathies such as upper and lower limb numbness and tingling (74). Chronic administration of AA to laboratory animals resulted in skeletal muscle weakness and ataxia (72, 75, 76). Numerous excellent reviews have covered in-depth literature on the neurotoxicity effects of AA (73, 77). The mechanism underlying the neurotoxicity of AA is inconclusive. However, several mechanisms were proposed to mediate the neurotoxic effects of AA. Interaction of AA with kinesin motor protein in neurons impaired fast anterograde transport of nerve growth factors from cell body to periphery, resulting in nerve death (60). AA was also shown to inhibit uptake of neurotransmitter into striatal synaptic vesicle through possible interaction with sulfhydryl groups on specific proteins and thereby disrupting presynaptic release of neurotransmitters (78).

### Reproductive Toxicity of AA

To date, reproductive toxicity of AA in humans is unheard of. In laboratory animals, exposure to high levels of AA proven to cause reproductive toxicity (79, 80). Various concentration range of AA and duration of AA exposure have been experimented on the reproductive toxic effects of AA in laboratory animals which resulted in decreased fertility rates, decreased litter sizes, abnormal sperm, decreased sperm counts, reduced copulatory activity, and decreased weight of testes (81–83). Further corroborating the reproductive toxicity of AA, Yilmaz et al. (84) investigated the effects of AA and GA exposure on mouse Leydig and Sertoli cells. The researchers showed exposure to AA and GA to reduce cell viability and increase oxidative stress and apoptosis in both Leydig and Sertoli cells (84).

Several theories were proposed to delineate the mechanistic effects of AA on reproductive toxicity. One of the theories relate the neurotoxic effects of AA to mating behaviors. AA-induced peripheral neuropathies such as reduced hind-limb function could impair copulatory behavior, mounting responses, and intromission (83), subsequently affecting proper deposition of sperm in the vagina and uterus and also the resulting hormonal changes (85). AA-induced inhibition of kinesin motor proteins, which are also found in flagella of sperm in addition to nervous system, could affect sperm motility and fertilization events (80, 86, 87). Regarding hormonal mechanism of action, AA reduced serum testosterone and prolactin levels (88) which could lead to testicular atrophy, affects sperm development and motility (81, 89).

### Immunotoxicity of AA

The literature on the immunotoxic potential of AA is limited. In rats, exposure to AA significantly decreased the weight of spleen, thymus, and mesenteric lymph nodes (90). Orally administered AA reduced alpha-naphthyl acetate esterase-positive lymphocytes counts and also caused lesions in ileal Peyer’s patches in a dose-dependent manner (91). In other words, AA was detrimental toward gut-associated lymphoid tissue and peripheral blood lymphocytes in rats (91). Fang et al. (92) reported decreased terminal body weight, thymus and spleen weights, and lymphocyte counts following exposure to AA in female BALB/c mice. The researchers also reported significant reduction in the percentage of natural killer cells, interleukin-6, and concanavalin A-induced splenocyte proliferation (92). Kim et al. (93) demonstrated AA-induced senescence in C57BL/6 male mice macrophages *via* ATF3-mediated pathway. The researchers also associated the AA-induced senescence to ROS production, activation of p38 and JNK signaling pathways, and also increased expression of p53 (Table 1) (93).

**Table 1:** Acrylamide (AA) exposure-induced toxicity.

Animal/cell lines	Route of administration/dosage	Biological samples/techniques/parameters assessed	Findings	Inference	Reference
Male wild-type (CYP2E1^+/+^) vs. CYP2E1-null mice (C57DL/6N2 × 129Sv), 3–4 months old	AA (0–50 mg/5 mL saline/kg), intraperitoneal	Plasma AA and GA levels; red blood cells—hemoglobin adducts; livers, lungs, testes—DNA adducts (LC–ES/MS/MS analysis)	AA produced significant increase in N7-GA-Gua and N3-GA-Ade adducts in all tissues of WT-mice. Significantly less adducts were detected in liver, lungs, and testes of mutant mice. Significant increase in AAVal-PTH and GAVal-PTH adducts in WT-mice. Ratio of GAVal-PTH: AAVal-PTH in AA-treated WT-mice was 1.7, the ratio was 0.02 in null mice	CYP2E1 is the main enzyme involved in epoxidation of AA to GA, which is responsible for formation of GA-DNA and hemoglobin adducts	(38, 39)

Human ovarian cancer cell lines PA-1, SK-OV-3, EF-27; human endometrial cancer cell lines MFE-319. FE-194; human primary hepatocytes	Incubation of cell lines with 0.02 and 1 mM AA and GA for 72 h	Microarray analysis, real-time PCR, Western blot analysis (genes or proteins involved in cellular stress, oncogenesis, xenobiotic metabolism)	Upregulation of the genes involved in angiogenesis such as TF3 (ovarian and endometrial cell lines), dual specific phosphatases DUSP1, DUSP4, and DUSP5 (ovarian cell lines), zinc finger protein gene ZNF746, cYMC and Bystin-like protein gene (BYSL) (primary human hepatocytes)	High dose of AA can induce genes with growth promoting potential like oncogene cMYC and genes involved in MAPK pathway	(41)

Male F344 rats	AA was administered in the drinking water at concentrations of 2.5, 10, and 50 mg/kg bw/day for 14 days	Motor activities; serum levels of thyroid-releasing hormone, thyroid-stimulating hormone (TSH), thyroid hormones (T3, T4); target tissue expression of genes involved in hormone synthesis, release, and receptors; dopamine and serotonin levels in hypothalamus and pituitary gland; and histopathological evaluation of the pituitary glands	50 mg/kg/day AA caused lethargy and hind-limb paralysis; 50 mg/kg/day AA significantly decreased serum T4 levels. No significant effects on other parameters assessed	Sub-chronic treatment of F344 male rats with AA provided no evidence for anti-thyroid effects manifested by compensatory increases in cell proliferation through dysregulation in the hypothalamus or pituitary	(49)

Male F344/DuCrl rats	0.0, 0.5, 1.5, 3.0, 6.0, or 12.0 mg AA/kg bw/day was given in drinking water for 5, 15, or 31 days	Expression profiling by next-generation sequencing of RNA, bioinformatics to identify differentially expressed genes and gene ontology pathway analysis, and qPCR was performed on testes samples	The largest number of differentially expressed genes (DEG) (65 transcripts) was observed at the highest AA exposure level (12.0 mg/kg/day) on day 31. 6.0 and 12.0 mg/kg of AA significantly increased Cyp2a1 rat testosterone 7 a-hydroxylase; QPCR showed significant increase in *Arsi* at 0.5, 1.5, 6.0, and 12.0 mg/kg, *Cacnb1*at 1.5 and 6.0 mg/kg, *Cacng7* at 0.5 and 12.0 mg/kg, *S100b* at 0.5, 1.5, 3.0, 6.0, and 12.0 mg/kg, *Tnnt2* at 12.0 mg/kg, *Trpc1* at 1.5 and 3.0 mg/kg (*p* ≤ 0.05)	AA *via* calcium signaling and cytoskeletal actin filaments can cause rat dominant lethal mutations that could lead to impaired chromosome segregation during cell division	(53)

Bovine eyes (3–4 years old)	Incubation of cell lines with staurosporine with/without 5 mM AA for 8 or 24 h; cells were preincubated with AA 5 mM in serum-containing medium; cells were incubated with AA 5 mM alone	Indirect immunofluorescence on antibodies against vimentin and human α-tubulin; percentage of apoptosis was calculated upon exposure to AA	AA exposure caused collapse of vimentin and microtubules paralleling cytoskeletal disruption; Cell adopted nearly a rounded morphology; thick f-actin bundles remaining in the cell periphery; AA slightly increased apoptosis compared to controls. Simultaneous exposure to AA and staurosporine for 8 h produced significantly less apoptosis and preincubation with AA followed by staurosporine reduced apoptosis at 8 and 24 h of treatment	AA exerts significant effects on the cytoskeleton of bovine eyes cells and AA can significantly attenuate the apoptotic effect of staurosporine	(56)

Male F344/DuCrl rats (8 weeks old)	Rats were given 0.0, 0.5, 1.5, 3.0, 6.0, or 12.0 mg AA/kg bw day in drinking water for 5, 15, or 31 days	Serum analysis of hormones [triidothyronine (T3), thyroxine (T4), reverse T3, TSH, lutenizing hormone, prolactin, and testosterone]; Ion Proton™ sequencing (RNA-seq) was done to calculate DEG and qPCR to quantitate gene expressions in liver and thyroid samples	6.0 mg/kg bw day AA for 31 days significantly increased the absolute and relative thyroid weights; five 5 days of exposure to 1.5 mg/kg bw day AA significantly decreased serum T3 levels. T4 levels were significantly increased on day 5 at the highest dose, and on day 31; gene expression in liver: high dose of AA affected gene expression of Brca1, Top2a, Cenpf, Nr1d1; gene expression in thyroid: Nqo1 and Hmox1 was differentially expressed at the highest dose on day 31, increased expression of Cyp2e1 on day 31 following exposure to 0.5 and 12 mg/kg bw day AA, genes involved in cytoskeletal pathways were differentially expressed (Acta1, Atp2a1, Myl1, Myh1, and Pvalb)	Mechanism of action for AA-induced thyroid carcinogenicity in male rats may due to perturbation of calcium signaling	(55)

Male Sprague-Dawley rats, 8 weeks old	Experiment 1: animals were gavaged (0, 30, 45, and 60 mg/kg/bw of AA) one a day for five consecutive days; Experiment 2: animals were gavaged single dose of 125, 150, and 175 mg/kg/bw of AA	Histochemical demonstration of alpha-naphthyl acetate esterase (ANAE) was done on blood and lymphoid tissue samples from the ileal Peyer’s patches (IPP)	Histopathology: 125, 150, and 175 mg/kg/bw of AA significantly reduced IPP size, depleted lymphoid cells in follicles of IPP, regression and reduction of germical left’s size; ANAE histochemistry: ANAE-positive lymphocyte levels decreased significantly (*p* < 0.05) in all AA received animals in a dose-dependent manner—highest decrease seen in 45 and 60 mg/kg/bw (Experiment 1), highest decrease seen in 150 and 175 mg/kg/bw (Experiment 2)	Exposure to AA is detrimental to peripheral blood lymphocytes and gut-associated lymphoid tissues in rats	(91)

Female BALB/c Mice (6–8 weeks old) of specific pathogen-free grade	4, 12, 36 mg/kg/bw of AA dissolved in distilled water given for 30 days	Assessment of body weight, spleen and thymus weight, hematological parameters, phenotyping of peripheral blood samples, quantification of serum cytokines and immunoglobulin, hemolysis test; plaque-forming cell assay and mitogen-induced splenocyte proliferation and splenic natural killer (NK) activity was assessed using mice splenic suspension; histopathology of various organs was studied	AA exposure significantly reduced body weight, spleen weight and thymus weight of mice; AA significantly increased percentage of T lymphocytes (CD^3+^, CD^19−)^, CD4^+^ T lymphocytes (CD^3+^, CD^4+^, Th cells); AA significantly decreased percentage of NK cells (CD^3−^, CD^49+)^; AA significantly reduced serum IL-6 level; AA significantly reduced the HC50 and the ConA-induced splenocyte proliferation; histopathology: mild atrophy of thymus, decrease in the number of bone marrow (hematopoietic) stem cells, shrinking of lymphoid nodules in germinal left in spleen, and white pulp atrophy of spleen, lymphopenia, and proliferation of fibrous tissue in lymph glands, unclearness or disappearance of follicle structure, and atrophy of lymph glands	AA inhibited cellular and humoral immunity of mice following 30 days of exposure	(92)

C57BL/6 male mice (young 7 weeks; aged 18−20 months)	RAW 264.7 cells and peritoneal macrophages were exposed to AA (<1 mM)	Macrophages tested in senescence associated-β-galactosidase (SA-β-gal) assay, telomerase repeat amplification protocol (TRAP) assay, cell cycle analysis, ROS production assay, ATF3 protein, and gene expression studies	AA (<1 mM) caused senescence-like growth arrest, significantly increased protein and gene expression of ATF3; intracellular ROS levels significantly increased in a time-dependent manner at 0.25 and 0.5 mM ACR; AA-induced macrophage senescence *via* the p38 and JNK pathways mediated by ATF3	ACR-induced senescence aided by ATF3 by enhancing ROS production, activating p38 and JNK kinases, and upregulating the expression of p53, collectively leading to cellular senescence in macrophages	(93, 94)

Mouse Leydig (TM3) and Sertoli (TM4) cell lines derived from the testis of immature BALB/c mice (11–13 days old)	Cell lines exposed to AA (10 and 1 mM) and GA (1 and 0.5 mM) for 24 h	Leydig and Sertoli cells were evaluated for cell viability, lactate dehydrogenase activities, lipid peroxidation, H_2_O_2_, apoptosis/necrosis rate, and mRNA levels of apoptotic genes	High AA and GA doses significantly decreased viability of Leydig cell Sertoli cells (*p* < 0.01); dose-dependent decrease in LDH activities of Leydig and Sertoli cells following AA and Ga exposure (*p* < 0.01); Leydig cells: significant increase in MDA levels in the all AA- and GA-treated groups. Sertoli cells: increase in MDA levels only by high AA and GA doses (*p* < 0.001); significant increases in H_2_O_2_ levels following AA and GA exposure in Leydig and Sertoli cells; 10 mM AA exposure of TM3 cell line significantly increased the expression of caspase3 (2.6-fold), Bax (1.3-fold), and p53 (1.1-fold) and decreased Bcl-2 (0.5-fold); 1 mM of AA increased the expression of caspase3 (2.5-fold), Bax (2.3-fold), and p53 (2.5-fold) and decreased Bcl-2 (0.3-fold); exposure of 1 mM GA to TM3 cell line, significantly increased caspase3 (2.1-fold), Bax (2.2-fold), and p53 (1.4-fold) and decreased the expression of Bcl-2 (0.5-fold) gene expression; GA (0.5 mM) increased caspase3 (3.6-fold), Bax (4.6-fold), and p53 (4.9-fold) and decreased Bcl-2 (0.2-fold)	Oxidative stress could play central role in AA and GA-induced apoptosis of Leydig and Sertoli cells	(84)

Adult male Sprague-Dawley rats	exposed to ACR at daily dose-rates of either 50 mg/kg/day (ip) for 5 days or 21 mg/kg/day (po) for 21 days	Body weight and gait score was determined; striatal synaptic vesicles from the rats were used to test for vesicular ^3^H-dopamine uptake and spontaneous efflux and density of degeneration was determined using amino-cupric silver staining and light microscopic examination; synaptosomal dopamine uptake and release following AA exposure (*in vivo*) was assessed; synaptosomal gluthathione content was assessed following AA exposure (*in vivo*)	50 mg/kg of AA reduced the body weight and increased the gait score; 21 mg/kg AA increased the gait score; exposure to 50 mg/kg/day × 5 days or 21 mg/kg/day × 21 days significantly decreased vesicular DA uptake and also reduced KCl-evoked synaptosomal DA release; AA did not affect neurotransmitter retention	AA impaired neurotransmitter uptake into striatal synaptic vesicles through interaction with sulfhydryl groups on functionally relevant proteins which leads to defective presynaptic release	(78)

### Hepatotoxicity of AA

Similar to the reproductive toxicity, there were no reports of AA hepatotoxicity in humans, although AA metabolism takes place in the liver. However, numerous studies have reported the harmful effect of dietary AA in the liver of experimental animals due to oxidative stress (95, 96). A high dose of 25 mg/kg AA administrated for 21 days resulted in significant decrease of liver GSH level and total antioxidant status in experimental adult rats (95). AA administration also lead to decrease of liver enzymes (e.g., aspartate aminotransferase, alanine aminotransferase, and alkaline phosphatase), superoxide dismutase, catalase activities, while total oxidant status and malondialdehyde levels increased (95). Moreover, the total cholesterol, triglyceride, and low-density lipoprotein cholesterol levels were increased, while high-density lipoprotein cholesterol was decreased following AA administration (96). Waterborne exposure of zebrafish in water containing AA (300 ppm) resulted in acute death within 26 h along with elevation of blood glucose, triglyceride, hepatic inflammation, and fat accumulation in the liver (94).

## AA Poisoning: Present and Future Mitigation Strategies

In the year 2005, European Food Safety Authority and the Joint Food and Agriculture Organization of the United Nations (FAO)/World Health Organization committee recommended to employ “margin of exposure (MOE)” approach to evaluate the risk assessment of AA (7, 97). The numerical value of MOE represents the ratio between the dose–response curve results in tumors in experimental animals and human dietary intake. Based on the modified risk assessment, the MOE value of AA showed the chemical to impose a severe human health concern compared to other chemicals, even the likes of benzopyrene (98).

Numerous strategies were shown to be able to inhibit or mitigate AA formation, both in model systems and actual food systems. These strategies include pH modification, decreasing the heating temperature and time, choosing raw materials with fewer precursors, adding the different exogenous additives, and process technology interventions (e.g., acids, amino acids, hydrogen carbonates, proteins, or antioxidant) (99, 100).

The AA formation in bread are significantly influenced by the yeast amount, fermentation time, fermentation temperature, and yeast types used in bread baking (101). Treatment with asparaginase produced from *Cladosporium* sp. (to convert the precursor asparagine to aspartic acid) to the wheat-based dough were able to reduce 97 and 73% of AA formation in the crust and crumb regions of the bread (102). Similarly, the use of asparaginase was able to cause 90% reduction in AA content after using a combination of asparaginase treatment and blanching of potato slices (103). In a separate study, asparaginase was also experimented on chilled French fries which reduced the AA content in the fries up to 90% without affecting final taste of the products (104).

Numerous studies used antioxidants as additives to inhibit AA formation. The inhibition of AA formation was in correlation with antioxidant activity of the additives (105). The addition of flavonoids significantly inhibited AA formation during microwave heat processing, where the inhibition strength were shown to be related to the number of phenolic hydroxyls of flavonoids (105). In addition, phenolic antioxidants also demonstrated an inhibiting effect in AA formation, with the most significant reductions (≈60%) observed for caffeoylquinic acids (100). Alternatively, addition of natural herbal extract also demonstrated inhibition of AA formation. Addition of antioxidants of bamboo leaves (AOB) and acylated AOB were able to reduce AA formation in fried potato crisps ranging from 30.7 to 46.9% (106). Similarly, the addition of green tea extract into burgers and nuggets coating were able to reduce AA formation (107).

## Summary

The research studies conducted on AA described the potential health risk of fried and baked carbohydrate-rich foods. There is clear evidence to show that AA is carcinogenic in rodents. However, there is paucity of facts depicting the health risk in humans as evident from numerous epidermiological and toxicological studies. There are lacunae in the past studies, especially with regard to lack of findings in estimating dietary AA intake through questionnaires and AA content database in foods, lack of repeated exposure estimations, and lack of statistical power to detect small increases in risk. Nevertheless, it is important that efforts should continue to reduce AA levels in food products. Further studies are suggested in human beings to show the detrimental effect of AA.

## Author Contributions

Conceptual framework and design: SD. Searched references: ST and JK. Drafted manuscript: ST, SD, and JK. Critically revised the manuscript: ST, SD, and JK.

## Conflict of Interest Statement

The authors declare that the research was conducted in the absence of any commercial or financial relationships that could be construed as a potential conflict of interest.
